# Enhancing diabetic rat peripheral nerve conduction velocity parameters through the potent effects of MitoTEMPO

**DOI:** 10.1016/j.heliyon.2024.e41045

**Published:** 2024-12-06

**Authors:** Murat Cenk Celen, Seckin Tuncer, Ahmet Akkoca, Nizamettin Dalkilic

**Affiliations:** a Ankara Medipol University, Faculty of Medicine, Department of Biophysics, Ankara, Türkiye; b Eskisehir Osmangazi University, Faculty of Medicine, Department of Biophysics, Eskisehir, Türkiye; c Selcuk University, Taskent Vocational School, Department of Occupational Health and Safety, Konya, Türkiye; d Baskent University, Faculty of Medicine, Department of Biophysics, Ankara, Türkiye

**Keywords:** MitoTEMPO, Nerve conduction velocity studies, Diabetic neuropathy, Streptozotocin

## Abstract

The efficacy of MitoTEMPO, a mitochondria-targeted antioxidant, in altering nerve fiber conduction properties within the sciatic nerve of streptozotocin (STZ)-induced diabetic rats, a model for diabetic neuropathy characterized by myelinated fiber atrophy and nodal abnormalities. Utilizing the STZ-induced diabetic rat model, we assessed the impact of MitoTEMPO on nerve function through compound action potential (CAP) analysis and histological evaluation. Key indicators such as maximum depolarization (MD), CAP area, and conduction velocity distribution (CVD) were measured to gauge MitoTEMPO's neuroprotective effects, alongside physical parameters like weight and blood glucose levels. MitoTEMPO treatment significantly preserved CAP parameters (MD and CAP area), indicative of its protective role against diabetes-induced nerve dysfunction. Moreover, it mitigated the diabetes-induced disruption in CVD, suggesting a preservation of nerve fiber activity. Histological analyses corroborated these electrophysiological findings, showing reduced damage in MitoTEMPO-treated nerves, such as thinner perineurium and less myelin sheath degeneration. Our findings highlight MitoTEMPO's potential as a mitigative agent against diabetic neuropathy's detrimental effects on nerve structure and function. The study underscores the therapeutic promise of mitochondria-specific antioxidants in managing diabetic neuropathy, advocating for further clinical research to explore MitoTEMPO's applicability in novel treatment strategies for peripheral neuropathies.

## 1 Introduction

Diabetes mellitus, a multifaceted metabolic disorder, is delineated into type 1, characterized by inadequate insulin production, and type 2, marked by insulin resistance [1,2]. Prolonged diabetes precipitates a spectrum of acute and chronic sequelae, notably cardiomyopathy and diabetic neuropathy, the latter posing a risk for limb amputation [3]. Nerve conduction studies, leveraging compound action potentials (CAP), furnish nuanced insights into diabetic neuropathy among other conditions. The heterogeneous conduction velocities and characteristics within peripheral nerves underscore the significance of ascertaining conduction velocity and its distribution via CAP recordings [4,5]. The application of supramaximal stimuli in CAP analyses stands paramount for diagnosing peripheral nerve afflictions, facilitating studies on nerve growth, regeneration, and evaluating the dispersion of nerve conduction velocities [6].

Research employing animal models to investigate diabetes-induced complications is prevalent, with streptozotocin-induced diabetes serving as a common paradigm to emulate diabetic neuropathy through the manifestation of myelinated nerve fiber atrophy. Investigations into single nerve fiber configurations have unveiled nodal irregularities, including paranodal and segmental demyelination, alongside axonal degradation within this model's peripheral nerves [[7], [8], [9]]. Concurrently, a burgeoning body of evidence associates augmented free radical levels with diabetes pathogenesis, positioning mitochondria, the epicenter of oxidative phosphorylation, as a pivotal focal point for therapeutic intervention [10]. In this context, antioxidants have garnered attention for their disease-mitigating potential, with MitoTEMPO (2-(2,2,6,6-tetramethylpiperidin-1-oxyl-4-ylamino)-2-oxoethyl)triphenylphosphonium chloride), a synthetic mitochondria-targeted antioxidant, emerging as a compound of interest [[11], [12], [13]]. By binding to triphenylphosphonium, it aims directly at mitochondria, mitigating oxidative stress at its source, thereby offering substantial protection to cellular integrity [[14], [15], [16], [17]].

Despite the extensive documentation on MitoTEMPO's protective capabilities, its utility in addressing diabetic neuropathy remains unexplored. This study endeavors to bridge this gap by examining MitoTEMPO's efficacy as a mitochondria-specific antioxidant in mitigating diabetic neuropathy and its influence on the distribution of nerve conduction velocities in diabetic contexts.

## 2 Materials and methods

### 2.1 Animal preparation

Due to gender-dependent differences in the rat sciatic nerve fiber CVDs [18] only male Wistar Albino rats weighing 250–300 g (12–14 weeks old) were provided from KONUDAM (Necmettin Erbakan University Experimental Medicine and Application Center, Konya, Türkiye) for the study. All experimental procedures on animals were carried out in approved by Meram Medical Faculty Experimental Ethics Committee (Approval no: 17-005, Necmettin Erbakan University, Konya, Türkiye) which is subject to the principles of ARRIVE. Rats were housed in cages at ambient temperature and humidity on a 12/12-h light/dark cycle. All animals received food and water ad-libitum.

The study involved three groups: the control group (CON, n = 10), diabetic group (DM, n = 12), and MitoTEMPO-treated diabetic group (Mito, n = 12). Diabetes was induced in DM and Mito groups via a single intraperitoneal (i.p.) injection of 50 mg/kg streptozotocin (STZ) dissolved in 0.1 M sodium citrate (pH 4.5). One week post-injection, rats with blood glucose levels ≥300 mg/dl were considered diabetic. The Mito group received MitoTEMPO (Cat No. 1334850-99-5; Sigma-Aldrich, Germany) (0.7 mg/kg/day) by gavage starting one week after STZ induction, for four weeks [19].

### 2.2 Preparation of sciatic nerves and experimental setup

Dissecated sciatic nerves were carefully removed from the hind limbs of euthanized rats. Nerves were immediately placed in a recording chamber which experiments will be helded. Continuously modified Krebs solution (in mmol per liter: NaCl 119, KCl 4.8, CaCl_2_ 1.8, MgSO_4_ 1.2, KH_2_PO_4_ 1.2, NaHCO_3_ 20, and glucose 10, pH 7.4, and gassed with a mixture of 95% O_2_ and 5% CO_2_) was perfused and temperature was maintained at 37 ± 0.5 °C. This helps to create stable experimental setup.

### 2.3 CAP recording

Nerve stimulations were conducted from proximal ends of the nerve trunk through a stimulus isolation unit (Model SIU5; Grass Instruments Co., Quincy, Massachusetts) using a stimulator (Model S88K; Grass Instruments Co.). Square-wave pulses of supramaximal intensity (0.2 ms duration, and 1-Hz frequency) were used for nerve stimulation. CAP recordings were performed by using a suction electrode [20] placed at the tibial branch of the isolated nerve trunk. Amplified CAP signals (Model CP511 AC amplifier; Grass Instruments Co.) were digitized by using an A/D converter card (Model PCL 1710; Advantech, Taiwan) at a 40-KSPS (kilosamples/s) using the open-source CAP recording software RETICAP (http://icon.unrlabs.org/projects/reticap) produced in Necmettin Erbakan University Biophysics Lab. and stored on a hard disk for further analysis.

### 2.4 Analyzing procedure

To investigate the status of neural function for three groups, advanced mathematical procedures were conducted on all CAP recordings. All the recorded CAP signals were recalled, and the following analyzing procedures were conducted. Since areas (mV·ms) under the CAP and the maximum depolarization (MD) value (mV) are proportional to the number of excited nerve fibers in that nerve, these values were calculated [21]. Two different conduction velocities, (CVcap and CVpeak), were obtained. For this purpose, two-time differences (Δtcap and Δtpeak) were measured. Δtcap is the time delay between the moment the stimulus is delivered and the onset of CAP, and Δtpeak is the time delay between the moment the stimulus is delivered, and the CAP amplitude reached its maximum value. By doing this, we have calculated the conduction velocity of the fastest fibers (CVcap) and relatively slow conducting fibers (CVpeak). The maximum and minimum time derivatives of CAPs [(dV/dt)max and (dV/dt)min] relate the maximum and minimum rates of change in the rising and falling phases of the CAP with time and can be used as an index of the conduction activity of nerve fibers in a bundle, so (dV/dt)max and (dV/dt)min values were obtained.

Conduction velocity of nerve fibers varies linearly with the diameter of the fibers. Evaluation of the CVD is important both for clinical and basic research. A suitable mathematical model that is the non-invasive method is needed to estimate CVDs from CAPs recorded at certain distances from the stimulus site [22,23]. To obtain the individual nerve conduction group activity we estimate nerve conduction velocity histogram by using the model that we enhanced beforehand [20,24]. Our model based on the model proposed by Ref. [25] and it is sensitive mostly to myelinated fibers as it is in other model studies [26].

CAP can be expressed as Eq. (1):

$$CAP\left(t\right)=\sum_{i=1}^{N}w_{i}.f_{i}.\left(t-\tau_{i}\right)$$

where N is the number of fiber classes; w_i_ is the amplitude-weighting coefficient for class i; τ_i_ is the propagation delay for fibers in class i. To estimate the individual activities of nerve fiber groups from CAPs, the CVDs for all nerves of the CON, DM, and Mito groups were calculated regarding Eq. (1).The CVD histogram is divided into three subgroups, Slow (11–40 m/s), Medium (40–63 m/s), and Fast (63–74 m/s), since the visually augmented effect of diabetes on nerve conduction velocity (NCV) can be helpful for ease of interpretation.

### 2.5 Histological examination

After electrophysiological experiments, sciatic nerve tissues of rats were taken. The extracted tissues were fixed in 10 % neutral buffered formaldehyde solution for 72 h. Tissue fixation was prepared by standard graded alcohol followed by xylene fixation procedure. Tissues were blocked to obtain sections from the microtome. The paraffin blocks were allowed to cool in the refrigerator for some time to obtain a comfortable section with a microtome. The blocks were then placed in the microtome. To obtain the best image in a light microscopic examination, the thickness of the sections was set to 4 μm. Hematoxylin Eosin staining method, which is one of the commonly used methods, was used in some of the sections. Some of them were stained with Toluidine Blue to better asses possible demyelination [27].The stained preparations were examined under a Nikon DS-F12 (Japan) microscope, and the images were photographed.

### 2.6 Statistical analysis

Normality of data was tested with histograms and Kolmogorov-Smirnov test for the continuous variables. To compare the groups, one-way analysis of variance (ANOVA) followed by Duncan post hoc test were used for multiple comparisons when analysis of variance indicated significant results. All data were expressed as mean ± SEM (standard error of means) for each group and significance was assumed at p < 0.05. Data were presented as mean ± sem. Statistical analysis and curve-fitting procedures were performed using the GraphPad Prism 5.0 for Windows software.

## 3 Results

### 3.1 General findings

Measurements of blood glucose levels and body weights were systematically recorded throughout the study, with specific attention paid to the initial and final weeks. Notably, the CON group exhibited a 33 % increase in body weight by the 5th week, in contrast to a 9.7 % increase in the DM group and a 29.1 % increase in the Mito group. Furthermore, a pronounced rise in blood glucose levels was observed in the DM group (229.6 %) and the Mito group (118 %) by the 5th week, highlighting the metabolic impact of the diabetic condition and the intervention. Although blood glucose levels seem to have decreased in the DM group data from the third and fourth weeks, it was determined that this decrease was correlated with the decrease in body weight. There is a correlation within the other groups in the same weekly change. Represented in Table 1.

**Table 1:** The values of body weight (g) and blood glucose (mg/dL) of animals of the groups at weekly.

	Body weight (g)				
	Initial Values	1st week	2nd Week	3rd Week	4th Week
CON (n = 10)	227.0 ± 12.8	272.0 ± 14.9	287.5 ± 15.7	298.9 ± 15.9	312.0 ± 17.2
DM (n = 12)	235.0 ± 16.3	265.5 ± 21,6	271.1 ± 22.9	281.5 ± 23.5	258.9 ± 27.6
Mito (n = 12)	227.6 ± 12.8	267.5 ± 11.7	275.5 ± 10.0	287.4 ± 10.6	293.3 ± 10.4
	**Blood glucose (mg/dL)**				

CON (n = 10)	108.3 ± 2.9	102.9 ± 2.3	100.1 ± 4.1	97.9 ± 3.8	98.2 ± 2.3
DM (n = 12)	108.8 ± 4.3	328.8 ± 13.8	350.6 ± 38.8	393.9 ± 45.1	351.3 ± 24.7
Mito (n = 12)	105.0 ± 1.8	321.4 ± 13.1	287.0 ± 31.5	232.4 ± 27.2	235.6 ± 41.7

Values are given as mean ± sem. CON: Control, DM: Diabetus Mellitus, Mito: MitoTEMPO Group.

### 3.2 Compound action potential (CAP) parameters

**Fig. 1:**
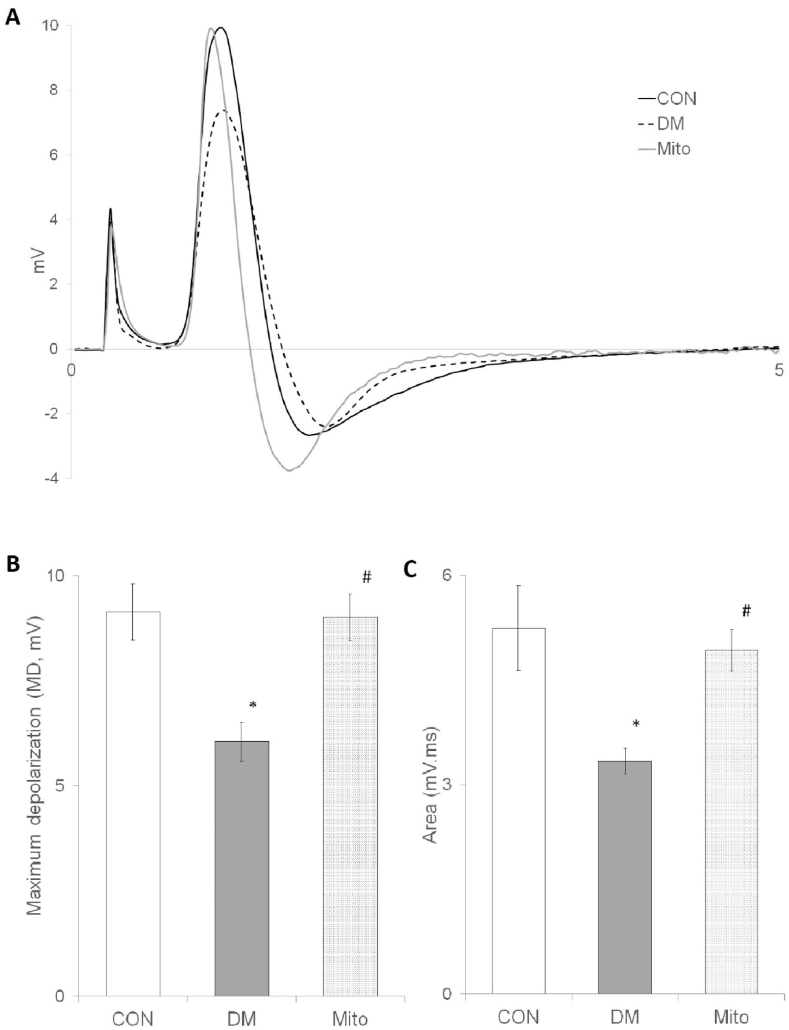
**(A)** Representative CAP samples that were recorded at the same distances by the suction electrode for CON, DM, and Mito group are shown in the same time axis with their pulse artifacts. MD values **(B)** and areas under CAPs **(C)** for each experimental group. ∗ shows significance when compared to CON while # shows significance between DM (p < 0.05). Values are given as mean ± sem.

Analysis of CAP traces illustrated distinct variations among the groups in Fig. 1A. A notable decrease in amplitude and a rightward shift were observed in the DM group compared to the CON group. Conversely, the Mito group demonstrated a preservation of amplitude with a leftward shift, suggesting differential impacts on nerve function. Figures representing these changes clearly indicate significant alterations in maximum depolarization (Fig. 1B) and CAP area among the groups (Fig. 1C), with the Mito group showing signs of recovery.

CV, an indicator of nerve fiber activity, was assessed for both latencies (CVcap and CVpeak) across all experimental groups. Comparative analysis revealed no significant differences in CV values between the DM and Mito groups when compared to the CON group, suggesting a maintained functionality in nerve conduction despite the diabetic condition and subsequent treatment. Time to peak (TP) values, indicative of the speed in the CAP's rising phase, were increased in both DM and Mito groups, though not significantly (Table 2).

**Table 2:** Some of the CAP parameters of experimental groups.

	CV_cap_ (m/s)	CV_peak_ (m/s)	(dV/dt)_max_ (mV/ms)	(dV/dt)_min_ (mV/ms)	TP (ms)
**CON (n=10)**	59.31 ± 2.57	38.90 ± 1.21	51.19 ± 3.09	−32.56 ± 3.09	0.26 ± 0.01
**DM (n=12)**	57.51 ± 1.32	36.56 ± 1.22	34.32 ± 3.29∗	−23.19 ± 2.78∗	0.31 ± 0.02
**Mito (n=12)**	59.36 ± 2.18	37.49 ± 1.73	48.45 ± 4.15^#^	−34.30 ± 2.97^#^	0.31 ± 0.02

Values are given as mean ± sem. ∗ shows significance when compared to CON while # shows significance between DM (p < 0.05). CON: Control, DM: Diabetus Mellitus, Mito: MitoTEMPO Group.

The maximum and minimum time derivatives of CAP (dV/dt)max and (dV/dt)min, which provide insights into the activities of the fastest and slower-conducting nerve fibers, respectively, were analyzed. Significant reductions in both (dV/dt)max and (dV/dt)min were observed in the DM group compared to the CON and Mito groups, highlighting the impact of diabetes on nerve fiber function. The Mito group displayed decreases in these values relative to the CON group, though not to a significant extent, indicating a degree of preservation of nerve fiber activity.

### 3.3 Conduction velocity distribution (CVD) parameters

**Fig. 2:**
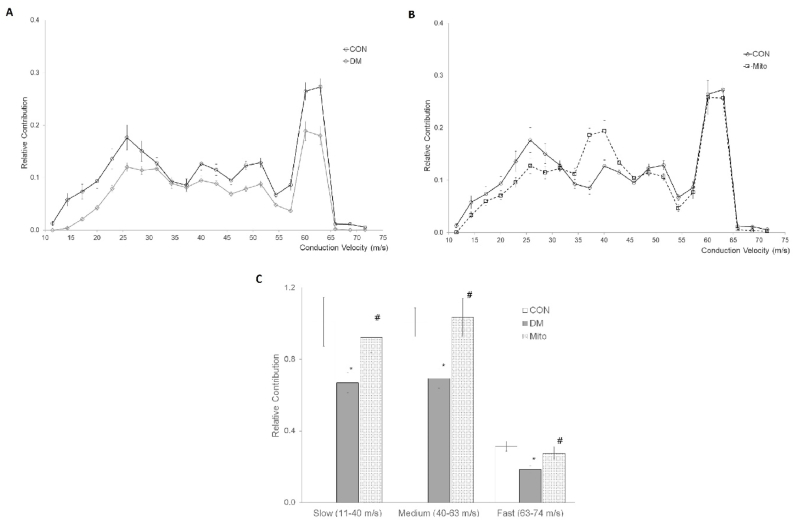
**(A)** The comparative CVD histograms of DM group with the CON group. Each value in the graph represents a percent relative number of fibers of that conduction velocity class. **(B)** The comparative CVD of the Mito group with the CON group. Each value in the graph represents a percent relative number of fibers of that conduction velocity class. **(C)** Recalculated conduction velocity distribution of three subgroups for three experimental groups of constituted conduction subgroups as described in the Methods (Slow:11–40 m/s; Medium:40–63 m/s; Fast: 63–74 m/s) for the groups CON, DM, and Mito. ∗ shows significance when compared to CON while # shows significance between DM (p < 0.05). Values are given as mean ± sem.

CVD analysis further delineated the effects of diabetes and MitoTEMPO treatment. Derived conduction velocity classes ranged from 11 m/s to 74 m/s across all groups. The relative fiber contribution of DM and Mito groups versus CON group is presented in Fig. 2A and B respectively. To facilitate interpretation, velocities were categorized into Slow (11–40 m/s), Medium (40–63 m/s), and Fast (63–74 m/s) subgroups. These subgroups revealed notable shifts in the distribution of nerve conduction velocities which are shown with bar graphs in Fig. 2C visually summarizing these changes for each experimental group.

### 3.4 Histological evaulations

**Fig. 3:**
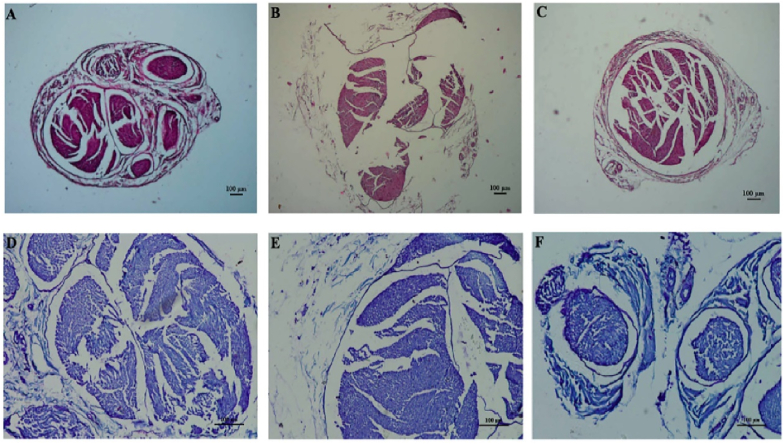
Light photomicrographs of histological cross-sections of the sciatic nerves samples belonging to the groups (100 μm). Image obtained by H&E of the sciatic nerve section for the CON group **(A)**, the DM group **(B)** and the Mito Group **(C)**. Image obtained by Toluidine Blue for the CON group **(D)**, the DM group **(E)** and the Mito group **(F)**.

Histological analysis provided visual confirmation of structural changes within the sciatic nerve. Light photomicrographs with Hematoxylin Eosin and Toluidine Blue in Fig. 3A, D depicted normal nerve structures in the CON group. In contrast, the DM group exhibited significant damage, including perineurium thinning and ruptures, myelin sheath degenerations, and endoneurium gaps in Fig. 3B–E. Notably, the Mito group showed histological improvements compared (Fig. 3C–F) to the DM group, with less degeneration and some thickening of the perineurium, suggesting a protective or reparative effect of MitoTEMPO on diabetic nerve damage.

## 4 Discussion

This investigation explored the potential of MitoTEMPO, a mitochondria-targeted antioxidant, in modulating the conduction properties of nerve fibers within the sciatic nerve of streptozotocin (STZ)-induced diabetic rats. The utilization of the STZ-induced diabetic rat model is a well-established approach for replicating the features of diabetic neuropathy, notably the atrophy of myelinated fibers, a hallmark of peripheral nerve damage in diabetes. Previous studies have documented the emergence of nodal abnormalities, including paranodal demyelination and axonal degeneration in this model, underscoring the deleterious impact of diabetes on nerve fiber integrity [8,9].

Diabetes (type 1) was produced by a one-time STZ injection was used. From the weekly physical parameters of rats’ findings (Table 1), at the end of the 5th week there was an increment in their weights; of the control group (CON group) by 37.4 % (85.9 g), of the diabetic group (DM group) by 10 % (23.9 g), and while that of the Diabetes + MitoTEMPO treated rats group (Mito group) by 28,9 % (65.7 g). Although blood glucose levels were not reduced to normal levels after four weeks of MitoTEMPO treatment, they remained significantly lower than those in the DM group. In other words, as the weeks passed, the percentage (%) increase in blood glucose levels was 225 for the DM group and 118 for the Mito group. These results show that MitoTEMPO reduces progression of diabetes. These findings are consistent with the literature [14].

Latency periods of three groups did not significantly differ which can be detectable even in representative traces in Fig. 1. The conventional CV estimations, commonly used in clinical and experimental studies are generally based on the measurements of latencies for calculating CV_cap_ and CV_peak_ of CAP signal [28,29]. We have observed that either measurement (Table 2) that represent activities of the fastest and the most contributing relatively to slow fibers in nerve bundle were not significantly different for both DM and Mito group compared to CON (p < 0.05). These findings might re-demonstrate that conventional conduction velocity measurement is insufficient in the early diagnosis of diabetes, and supports our findings suggested beforehand [5,24].

The MD value and the CAP area (Fig. 1B and C) for the Mito group did not change significantly when compared with CON group. These results might indicate the protective effect of MitoTEMPO on disturbing influence of diabetes. Since the time derivative of a function gives the rate of change of that function over time, the change in CAP form over time also gives information about the contribution of single nerve action potential (SNAP) in the CAP [30]. As can be seen from Table 2, diabetes alleviates the (dv/dt)_max_ and (dV/dt)_min_ values significantly as compared to CON and Mito group values, that values relate to the maximum and minimum rates of change in the rising and falling phases of the CAP with time [30,31]. These findings also show that while diabetes causes conduction blockage or slowing down in some type of fibers, MitoTEMPO protects the nerve from the disturbing effect of diabetes.

The CV is closely related with density of channels in the mambrene patch and passive membrane properties. A myelinated axon has ion channels, pumps and exchangers responsable for determining excitability. In the node of Ranvier, Na^+^ channels are densely clustered together with slow K^+^ channels, which contribute to the excitability and resting membrane potential [32]. The two passive membrane properties; time constant (τ = R_m_C_m_) that is designated by membrane capacity (C_m_) and membrane resistance (R_m_), and space constant (λ = √R_m_/R_o_, R_o_: extracellular resistance) are other factors that determine CVs. Consequently the evaluation of the distribution of a nerve fiber conduction velocity (CV) is important for the measurement of nerve function. Determination of te CVD is a numerical method and when applied to recorded CAP may gather information on the relative number of active fibers for discrete conduction velocity values in a nerve trunk [20,33]. Hence with CVD the functional state of a given fiber group may be monitored before and after a certain event. So making quantitative comperison between groups would be possible.

The CVD histogram findings suggest that diabetes decreased the relative contribution of fibers to all conduction velocity classifications as compared with those in the control group's histogram (shown in Fig. 2A and B). The total area under relative contribution (%) histogram relates to the total number of fibers in a nerve bundle. The area decrement for the DM group compared to the CON group was found to be 21.2 %. This indicates that 21.2 % of the total number of fibers was blocked in any way. The reason for this decrement may be interpreted as, by affecting conduction parameters of nerve fibers stated before, diabetes causes blockage or a slowdown in the conduction velocity of some of the fibers. When we compare the relative contribution graph of Mito group with CON group, there was also a decrement in area, but not as much as that in DM group (8.8 %). Specifically, there was no decrement in the relative contribution (%) of fast and fastest fiber classes. This result is an indication that MitoTEMPO reduces or protects the disruptive effect of diabetes.

The percentage contribution of the subgroups (Slow, Medium and Fast) obtained from the CVD histogram for CON, DM, and Mito groups may give more concrete results. The relative contribution (%) for the DM group in all three subgroups were found to be decreased significantly (p < 0.05). Decrement for Slow subgroup was 40 % compared to CON, and 33 % compared to Mito; for the Medium group, it was %40 compared to CON and 42 % compared to Mito; for the Fast subgroup, it was 33 % compared to CON and 20 % compared to Mito. When we compare all three subgroup findings that were calculated for the Mito group with CON subgroup findings, no significant difference has been found (p < 0.05). The fact that fiber loss is not significant in the Mito group indicates that MitoTEMPO has a significant protective effect on diabetes. These results are also consistent with the findings of other researchers [34,35]. These results also show that the CVD method is a powerful means for evaluating the status of any fiber class in a nerve. When histological findings are evaluated, it has been seen those negative effects of diabetes such as significant damages in the perineurium, thinning, ruptures and degenerations in myelin sheaths were relatively less observed in nerve sections of Mito group (Fig. 3). Histological findings have also shown that MitoTEMPO suppresses the negative effect of diabetes on nerve fibers. Histological findings also support our calculated electrophysiological results.

There are some limitations to this study. To offer insights into potential causes of observed diabetes related changes, food and water intake measurements should be followed during the study. Another limitation could be that the tendency of MitoTEMPO treatment to lower blood glucose levels could have been followed over a longer period of weeks, so that it could have been determined whether these levels had returned to normal blood glucose levels at some point. In addition, the fact that oxidative stress parameters could not be examined in the study is seen as a limitation. High quality histological images might also be helpful for detailed analysis of the effect of diabetes and the therapeutic agent MitoTEMPO.

In conclusion, our study not only confirms the detrimental effects of diabetes on nerve fiber conduction and structure but also underscores the potential of MitoTEMPO as a protective agent against these changes. The consistency between electrophysiological and histological outcomes reinforces the notion that mitochondria-specific antioxidants could play a crucial role in managing diabetic neuropathy. Further research is warranted to explore the clinical applicability of these findings, potentially leading to novel therapeutic strategies for peripheral neuropathies.

## CRediT authorship contribution statement

**Murat Cenk Celen:** Writing – review & editing, Writing – original draft, Visualization, Resources, Methodology, Investigation, Formal analysis, Data curation, Conceptualization. **Seckin Tuncer:** Writing – review & editing, Writing – original draft, Visualization, Methodology, Conceptualization. **Ahmet Akkoca:** Writing – review & editing, Writing – original draft, Visualization, Methodology, Formal analysis, Data curation, Conceptualization. **Nizamettin Dalkilic:** Writing – review & editing, Writing – original draft, Supervision, Resources, Project administration, Investigation, Conceptualization.

## Data availability statement

Dataset is available at the Mendeley under https://data.mendeley.com/datasets/hb874p4jkh/1 link.

## Declarations

All experimental procedures on animals were carried out in approved by Meram Medical Faculty Experimental Ethics Committee (Approval no: 17-005, Necmettin Erbakan University, Konya, Türkiye) which is subject to the principles of ARRIVE.

The authors of this manuscript declare that

the work described has not been published previously.

the article is not under consideration for publication elsewhere.

the article's publication is approved by all authors and tacitly or explicitly by the responsible authorities where the work was carried out.

if accepted, the article will not be published elsewhere in the same form, in English or in any other language, including electronically without the written consent of the copyright-holder.

## Funding

This study was supported by 10.13039/501100016981Necmettin Erbakan University Scientific Research Projects Funds, Türkiye with the Project number 171218007.

## Declaration of competing interest

The authors declare that they have no known competing financial interests or personal relationships that could have appeared to influence the work reported in this paper.
